# What else can we do to prevent diabetic retinopathy?

**DOI:** 10.1007/s00125-023-05940-5

**Published:** 2023-06-06

**Authors:** Rafael Simó, Cristina Hernández

**Affiliations:** 1 Department of Medicine, Universitat Autònoma de Barcelona, Barcelona, Spain; 2 Vall d’Hebron Research Institute (VHIR), Vall d’Hebron University Hospital, Barcelona, Spain; 3 CIBER of Diabetes and Associated Metabolic Diseases (CIBERDEM, ID CB15/00071), Instituto de Salud Carlos III (ISCIII), Madrid, Spain

**Keywords:** Diabetic retinopathy, Modifiable risk factors, Neurovascular unit

## Abstract

The classical modifiable factors associated with the onset and progression of diabetic retinopathy are the suboptimal control of blood glucose levels and hypertension, as well as dyslipidaemia. However, there are other less recognised modifiable factors that can play a relevant role, such as the presence of obesity or the abnormal distribution of adipose tissue, and others related to lifestyle such as the type of diet, vitamin intake, exercise, smoking and sunlight exposure. In this article we revisit the prevention of diabetic retinopathy based on modulating the modifiable risk factors, as well as commenting on the potential impact of glucose-lowering drugs on the condition. The emerging concept that neurodegeneration is an early event in the development of diabetic retinopathy points to neuroprotection as a potential therapeutic strategy to prevent the advanced stages of the disease. In this regard, the better phenotyping of very early stages of diabetic retinopathy and the opportunity of arresting its progression using treatments targeting the neurovascular unit (NVU) are discussed.


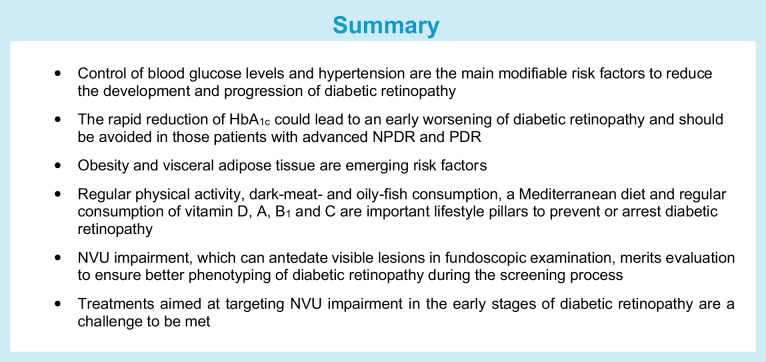


## Introduction

Diabetic retinopathy remains a leading cause of preventable visual impairment and blindness in the working age population [1]. In addition, it is one of the most common and most feared complications of diabetes, which imposes a severe burden on patients, healthcare systems and society throughout the world. In addition to controlling the modifiable risk factors, current treatment is based on laser photocoagulation and intravitreal injections of glucocorticoids, anti-vascular endothelial growth factor (VEGF) drugs or other anti-angiogenic agents [2]. These treatments are aggressive, with significant side effects, and are used to prevent severe visual impairment and blindness in the very advanced stages of the disease. Over the last decade, the concept that neurodegeneration is an early event in the pathogenesis of diabetic retinopathy has strongly emerged and, therefore, strategies based on neuroprotection seem a promising new avenue for treating the early stages of the condition [2, 3].

In this article the prevention based on modulating the modifiable risk factors is revisited, and the potential impact of antidiabetic drugs on diabetic retinopathy is commented on. Finally, the opportunity of arresting diabetic retinopathy progression at very early stages based on treatments aimed at preventing or reversing the early impairment of the neurovascular unit (NVU) is discussed.

## Revisiting modifiable risk factors

The classical modifiable factors associated with the onset and progression of diabetic retinopathy are the suboptimal control of blood glucose levels and hypertension, as well as the presence of dyslipidaemia. However, there are other less recognised modifiable factors that can play a relevant role, such as the abnormal distribution of adipose tissue, as well as lifestyle considerations such as the type of diet, vitamin intake, exercise and smoking, among other factors.

### Glycaemic control

The most relevant risk factor for the development of diabetic retinopathy is suboptimal glycaemic control. There is robust evidence supporting the relationship between blood glucose levels and the development and progression of diabetic retinopathy [4, 5]. In fact, the higher the reduction of HbA_1c_ the higher the effect in preventing or arresting the development/progression of diabetic retinopathy. However, there is, as yet, no information regarding the long-term effect of blood glucose control on retinal neurodysfunction or neurodegeneration in terms of prevention or even potential regression.

The role of glycaemic variability on diabetic retinopathy is less clear but time in range has been associated with all the stages of diabetic retinopathy in type 2 diabetes [6]. In addition, a systematic review and meta-analysis showed that fasting plasma glucose variability was strongly associated with the risk of development and progression of diabetic retinopathy in type 2 diabetes [7]. Finally, HbA_1c_ variability, which reflects longer-term glucose variability, contributes to the risk of diabetic retinopathy in both type 1 and type 2 diabetes [8, 9]. On this basis, it could be postulated that glucose-lowering agents with more capacity to mitigate glucose variability, such as current long-acting insulins or the next generation of ‘smart insulin’, could reduce the risk of diabetic retinopathy. However, specific studies addressed to answer this question are needed.

The early worsening of diabetic retinopathy due to the rapid improvement of hyperglycaemia is a topic that has been revisited in recent years [10]. This has been found in both type 1 and type 2 diabetes and, in addition to reductions in glucose levels following treatment with insulin and other glucose-lowering drugs, it has also been reported after diet or bariatric surgery, thus supporting the concept that the velocity of blood glucose reduction is more important than any particular glucose-lowering strategy. The main factors associated with early worsening of diabetic retinopathy are the magnitude of the reduction in HbA_1c_ (i.e. HbA_1c_ reduction >1.5% within 3 months or >2% within 6 months) and the presence of pre-existing diabetic retinopathy, particularly pre-proliferative and proliferative diabetic retinopathy (PDR) [10, 11].

### Blood pressure

Several randomised controlled trials have demonstrated the benefits of blood pressure control as a major modifiable factor for diabetic retinopathy incidence and progression [12, 13]. Overall, the results of these studies suggest that, while uncontrolled hypertension could lead to the onset or progression of diabetic retinopathy, slight or mild hypertension does not significantly affect the condition.

Regarding the differential effect of anti-hypertensive drugs on diabetic retinopathy, there is only weak clinical evidence that ACE-i (ACE inhibitors) and angiotensin II receptor (ARA-II) antagonists exert additional beneficial effects in slowing diabetic retinopathy progression independently of their hypotensive properties [14, 15]. Therefore, the reduction of blood pressure is the key point, rather than the type of anti-hypertensive drug used to achieve this.

As is the case with metabolic control, there is no information yet regarding the effect of tight control of blood pressure on retinal neurodysfunction or neurodegeneration.

### Dyslipidaemia

Dyslipidaemia is generally considered to be a risk factor for diabetic retinopathy progression, but with less significance than hyperglycaemia or hypertension. It has been reported that statins reduce the incidence of hard exudates and microaneurysms, and decrease vision loss [16, 17]. In addition, fenofibrate prevents the progression of diabetic retinopathy [18], and the Action to Control Cardiovascular Risk in Diabetes (ACCORD)-EYE study demonstrated that the combination of fenofibrate and simvastatin diminished diabetic retinopathy by approximately 40% in comparison with participants taking simvastatin alone [19]. However, these results seem unrelated to the effects on lipids, and could be mainly accounted for by the anti-inflammatory and antioxidant actions of fenofibrate [3, 20].

### Obesity

A meta-analysis of prospective studies published in 2018 revealed that obesity increased the incidence of non-proliferative diabetic retinopathy (NPDR), but not PDR in individuals with type 2 diabetes [21]. In addition, studies performed in Asian populations indicate that isolated abdominal obesity (i.e. high waist circumference in the absence of obesity in terms of BMI) is associated with diabetic retinopathy [22, 23]. This finding is significant because it underlines the potential role of proinflammatory cytokines produced by adipocytes and macrophages infiltrating visceral adipose tissue on the pathogenesis of diabetic retinopathy.

Another obesity-related risk factor for diabetic retinopathy is neck circumference, a useful predictor of obstructive sleep apnoea [24], which in turn is associated with the presence and severity of diabetic retinopathy [25]. Individuals with diabetes who have a larger neck circumference are more likely not only to have diabetic retinopathy, but also to progress to more severe stages [26]. This is because the recurrent episodes of hypoxaemia due to apnoea stimulate angiogenic factors such as VEGF.

It should be noted that the new subclassification of individuals with adult-onset diabetes shows that Group 2, severe insulin-deficient diabetes (SIDD), which includes individuals with high HbA_1c_, impaired insulin secretion and moderate insulin resistance, have the highest incidence of diabetic retinopathy [27]. This finding suggests that insulin deficiency could play a key role in the pathogenesis of diabetic retinopathy and raises the question of whether SIDD patients would benefit for early intensive insulin therapy for the prevention of diabetic retinopathy.

### Lifestyle

#### Type of diet and vitamin intake

Fish consumption seems important in preventing or arresting diabetic retinopathy. In middle-aged and older individuals with type 2 diabetes, intake of at least 500 mg/day of dietary long-chain *n*-3 polyunsaturated fatty acids, easily achievable with two weekly servings of oily fish, is associated with a reduction in relative risk (48%) of sight-threatening diabetic retinopathy [28]. In addition, dark-meat fish (e.g. salmon, mackerel, swordfish, sardines or bluefish) consumed weekly (85–141 g) vs never was related to an almost 70% reduced likelihood of developing diabetic retinopathy [29].

A Mediterranean diet is also associated with prevention of diabetic retinopathy development. The PREvención con DIeta MEDiterránea (PREDIMED) study showed that a Mediterranean diet enhanced with extra virgin olive oil reduced the risk of incident diabetic retinopathy by 40% [30]. Extra virgin olive oil may be particularly beneficial because it is extracted from the olive fruit without using heat or chemical solvents. This mechanical process retains the highest amount of natural phenolic compounds, which have antioxidant and anti-inflammatory properties.

An important component of diet is the vitamin content. It has been consistently reported that adequate vitamin D status prevents the development of diabetic retinopathy [29]. In addition, several reports have found that individuals with diabetes present with lower levels of vitamin D than the non-diabetic population [31]. Therefore, it is important to monitor the vitamin D levels in patients with diabetes, not only for the prevention of osteoporosis but also diabetic retinopathy. Dark fish and fortified foods such as milk or cereals are important sources of vitamin D.

It is worth mentioning that vitamin D in the eye is locally produced, activated and regulated. In fact, the eye expresses all the enzymes involved in vitamin D_3_ production [32]. This is important because 1α,25-dihydroxyvitamin D_3_ inhibits angiogenesis and this effect is mediated by vitamin D receptors, which are also essential during retinal vascular development [33].

Vitamin A is essential in visual processes and is a main component of rhodopsin. So, the regular consumption of foods high in vitamin A or carotenoid precursors, such as kale, spinach, broccoli, carrots or sweet potatoes, is recommended. Vitamin B_1_ is also a potent free radical scavenger that prevents activation of the polyol pathway. Fortified breakfast cereals are an important source of vitamin B_1_. Finally, vitamin C (ascorbic acid) is also involved as a protective factor against diabetic retinopathy development. Vitamin C has antioxidant and anti-angiogenic actions and improves endothelial function. In 2010, we reported that patients with PDR presented lower intravitreous levels of ascorbic acid than non-diabetic individuals [34], which was confirmed more recently by other authors [35]. This is because ascorbic acid and glucose compete to use GLUT-1 to reach the retina and, therefore, the higher the glucose levels, the lower the uptake of ascorbic acid by the retina. So, apart from recommending a regular consumption of foods with high content of vitamin C, such as citrus fruits, good glycaemic control is also essential to maintain correct intraretinal levels of ascorbic acid.

#### Smoking

Another factor related to lifestyle is tobacco smoking. There is controversial evidence regarding smoking as a risk factor for the development or progression of diabetic retinopathy. Notably, a recent study found an association between smoking habit and deleterious findings in angio-optical coherence tomography (OCT) in patients with no visible diabetic retinopathy, so in very early stages of the condition [36]. Although other studies have had conflicting results, this should not alter the message about the importance of smoking cessation.

#### Physical activity

As regards exercise, it has been reported that regular physical activity reduces the risk of developing diabetic retinopathy [37]. However, it should be underlined that individuals with PDR should avoid high-intensity aerobic and resistance exercise, and physical activities comprising Valsalva manoeuvres (which increase systolic blood pressure). This is to reduce the risk of vitreous haemorrhage and retinal detachment. Specific sports such as boxing, high altitude mountaineering and scuba diving should also be avoided in individuals with PDR.

#### Sunlight exposure

Another potential risk factor for developing diabetic retinopathy is related to the deleterious effect of ultraviolet B radiation on retinal pigment epithelium. Although a moderate exposure to sunlight is recommended to maintain circulating vitamin D levels, a recent study has shown that sunlight exposure for ≥5 h a day is significantly associated with an increased risk of diabetic retinopathy [38]. The underlying mechanism includes the accumulation of reactive oxygen species that can damage retinal blood vessels causing retinal capillary apoptosis, hypoxia and neovascularisation. This evidence suggests that reduction in sunlight exposure could be a preventive strategy against diabetic retinopathy in people with diabetes, and opens up the possibility of testing sunglasses as a tool for this purpose in appropriate clinical trials.

### Impact of glucose-lowering drugs on diabetic retinopathy

The strong relationship between the reduction of HbA_1c_ and the beneficial effects on diabetic retinopathy has obscured the necessity of performing clinical trials investigating the specific effects of glucose-lowering drugs per se on diabetic retinopathy, independently of their effectiveness in reducing blood glucose levels. Therefore, we do not have robust information on this issue. This is very different from the available information that we have regarding the effects of glucose-lowering drugs on cardiorenal events, which have been widely studied. For this reason, studies aimed at examining the specific role of glucose-lowering drugs on the development and progression of diabetic retinopathy are needed. To the best of our knowledge only one clinical trial is currently ongoing to examine this issue. This is the FOCUS study (NCT03811561) a Phase III randomised clinical trial which will look at the long-term effects of semaglutide on diabetic eye disease when compared with placebo. A total of 1500 participants with type 2 diabetes will be included and the estimated study completion date is November 2027.

## New insights and perspectives on diabetic retinopathy prevention based on targeting the NVU

The concept that neurodegeneration is an early event in the development of diabetic retinopathy, which antedates and participates in its pathogenesis, has led to neuroprotection as a potential therapeutic strategy to arrest the progression of the condition [3]. As is the case with the brain, retinal neurodegeneration is not an isolated neuronal process and relies on the complex impairment of all the components of the NVU: macro- and microglia, neurons and vascular components (endothelial cells and pericytes). Among these cells, the glial activation plays an essential role in linking neuronal damage with early vascular impairment, which comprises the breakdown of the blood–retinal barrier and, consequently, vascular leakage [39].

### Experimental evidence

One of the more logical strategies for treating diabetes-induced retinal neurodegeneration is ‘replacement’ treatment of the neurotrophic factors that are downregulated in the early stages of diabetic retinopathy, such as pigment epithelial derived factor, somatostatin and glucagon-like peptide 1 [3]. Treatment with these neurotrophic factors has prevented the development of diabetic retinopathy in experimental models. Dipeptidyl peptidase IV (DPP-IV) inhibitors have also provided beneficial effects on NVU [40]. Although the enhancement of intraretinal levels of GLP-1 seems a relevant mechanism accounting for the beneficial effects of DPP-IV inhibitors, the activation of other pathways related to DPP-IV inhibition cannot be ruled out. In addition, the lower cost and higher stability of DPP-IV inhibitors in comparison with GLP-1, could mean they are excellent candidates for clinical development. Presynaptic proteins, which are crucial for neurotransmission and synaptic homeostasis, as well as proteins involved in axonal transport, are also downregulated in the diabetic retina [41, 42]. Therefore, a replacement treatment or therapeutic strategies addressed to avoid the diabetes-induced intraretinal reduction of these neurotransmitters could exert beneficial effects. However, further research in this field is needed.

Another successful strategy has been to block the endothelin-1 receptors ETB-R and ETA-R. By blocking these receptors, bosentan administered using eye drops exerted a beneficial effect on both neurons (blockade of ETB-R) and microvasculature (blockade of ETA-R), thus preventing retinal neurodegeneration and vascular leakage [43].

### Clinical perspectives

To the best of our knowledge, there are no drugs recommended by any scientific society aimed at targeting the NVU for treating the early stages of diabetic retinopathy. The usefulness of corticosteroids or non-steroidal anti-inflammatory drugs (NSAIDs) by topical route for treating diabetic macular oedema (DME) in humans have been reported [44, 45]. However, a lack of effect in reducing retinal thickness after 1 year of topical administration of the NSAID nepafenac has also been reported in patients with non-central-involved DME [46].

Fenofibrate and calcium dobesilate (two drugs administered orally) have shown to be effective and safe for the treatment of diabetic retinopathy in several clinical trials [3], but they are not formally recommended in clinical guidelines. It is worth mentioning that three large randomised clinical trials aimed at evaluating the effect of fenofibrate in arresting the progression of diabetic retinopathy are ongoing in the USA (NCT04661358), Australia (NCT01320345) and UK (Scotland; NCT03439345). These studies will provide new evidence on the usefulness and safety of fenofibrate for treating the early stages of diabetic retinopathy and could be helpful to cover the treatment gap that currently exists for early-stage diabetic retinopathy.

However, the long-term systemic administration of drugs has two main problems. First, they need to be able to cross the blood–retinal barrier, which could be a limiting factor to reach the retina at pharmacological concentrations. Second, systemic adverse effects and potential pharmacological interferences with other drugs used for the treatment of diabetes and its comorbidities is also a drawback that needs to be considered. In addition, the use of repeated intravitreal injections seems a strategy disproportionally aggressive for treating the early stages of diabetic retinopathy. For these reasons, topical treatment (eye drops) targeting the NVU has emerged as a new strategy for treating the early stages of the condition [2, 3]. However, only somatostatin has so far been tested in a randomised clinical trial (the European Consortium for the Early Treatment of Diabetic Retinopathy [EUROCONDOR] study). Although the results of this study were positive in terms of preventing the progression of neurodysfunction, there was no impact on microvascular damage [47]. This could be attributed to the high proportion of participants included in the study with no or very mild microvascular disease, the excellent metabolic control throughout the study and the short follow-up (2 years).

**Fig. 1:**
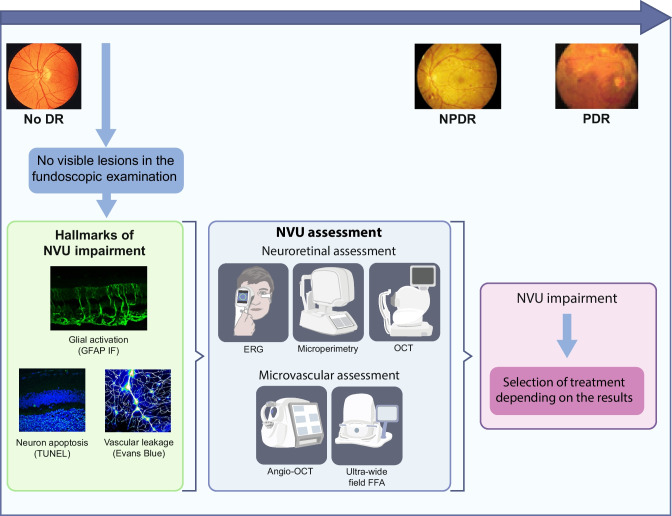
In the early stages of diabetic retinopathy, even before microvascular abnormalities can be detected in funduscopic examination, NVU impairment is already present in a significant proportion of patients. The main hallmarks of this diabetes-induced NVU impairment are: glial activation (also named reactive gliosis), neuron apoptosis and vascular leakage due to the disruption of the blood–retinal barrier. At this stage, neuronal and microvascular assessment would show us the degree of neural and/or microvascular damage, thus allowing a better phenotyping. This information would be important for predicting the risk of cardiovascular events and cognitive decline, as well as to select the most appropriate treatment for the early stages of diabetic retinopathy, when such treatments are available. DR, diabetic retinopathy; GFAP, glial fibrillary acidic protein; IF, immunofluorescence

An important lesson from the EUROCONDOR study is that a significant proportion (35%) of individuals with type 2 diabetes have early microvascular disease without detectable neurodysfunction [47]. Thus, neurodysfunction or neurodegeneration are not always the first abnormalities that occur in early stages of diabetic retinopathy. This finding underlines the need to incorporate the assessment of NVU integrity for identifying those patients in whom neuroprotective treatment might be of higher benefit (Fig. 1). Therefore, methods for the assessment of neurodysfunction, such as microperimetry or flicker electroretinogram (ERG) handheld recording device (RETeval), or methods such as OCT allowing the assessment of structural damage (e.g. neuroretinal thinning), should be included in the screening of diabetic retinopathy [40]. In addition, angio-OCT and ultra-wide field fundus fluorescein angiography (FFA) will improve our understanding of microvascular impairment. These examinations could be performed even when there are no visible lesions in fundoscopic examination. It seems clear that the development and implementation of this new and more complex screening will depend on the appearance in the market of effective and safe drugs for treating early-stage diabetic retinopathy. In this regard, clinical trials of drugs with experimental effectiveness in preventing both neurodegeneration and vascular leakage, such as GLP-1, DPP-IV inhibitors or endothelin blockers, are needed [2, 3]. In the meantime, given the high risk of cardiovascular disease and dementia that is present among the type 2 diabetic population with diabetic retinopathy, the better phenotyping of this condition will permit us to identify individuals more prone to developing either cardiovascular events or cognitive impairment [48, 49]. In addition, since retinal neuron loss is related to deficient sensory capacity and vision-related quality of life, periodic assessments of neurodegeneration/neurodysfunction in the diabetic population seems warranted [39].

In summary, apart from controlling modifiable risk factors, the early identification of patients with diabetes with NVU impairment could revolutionise the current management of diabetic retinopathy (see Summary Text box). At present, a transoceanic taskforce funded by JDRF and The Mary Tyler Moore & S. Robert Levine Charitable Foundation has been created to change the evaluation and grading of diabetic retinopathy based on this new evidence [50]. This initiative would be the beginning of a new era in the diagnosis and treatment of diabetic retinopathy, thus allowing better phenotyping and, consequently, a more personalised and cost-effectiveness treatment.

## Abbreviations

DME: Diabetic macular oedema
DPP-IV: Dipeptidyl peptidase IV
ERG: Electroretinogram
EUROCONDOR: European Consortium for the Early Treatment of Diabetic Retinopathy
FFA: Fundus fluorescein angiography
NVU: Neurovascular unit
NPDR: Non-proliferative diabetic retinopathy
NSAID: Non-steroidal anti-inflammatory drugs
OCT: Optical coherence tomography
PDR: Proliferative diabetic retinopathy
SIDD: Severe insulin-deficient diabetes
VEGF: Vascular endothelial growth factor
